# Immunoregulatory molecule expression on extracellular microvesicles in people living with HIV

**DOI:** 10.3389/fimmu.2024.1354065

**Published:** 2024-03-04

**Authors:** Deborah Neyrinck-Leglantier, Marie Tamagne, Raida Ben Rayana, Souganya Many, Paul Vingert, Julie LeGagneux, Adèle Silane Delorme, Muriel Andrieu, Eric Boilard, Fabrice Cognasse, Hind Hamzeh-Cognasse, Santiago Perez-Patrigeon, Jean-Daniel Lelievre, France Pirenne, Sébastien Gallien, Benoît Vingert

**Affiliations:** ^1^Univ Paris Est-Creteil (UPEC), Institut National de la Santé et de la Recherche Médicale (INSERM), Institut Mondor de la Recherche Biomédicale (IMRB), Creteil, France; ^2^Etablissement Français du Sang (EFS), Ivry-sur-Seine, France; ^3^Laboratory of Excellence, Biogénèse et Pathologies du Globule Rouge (GR-Ex), Paris, France; ^4^Service de Maladies Infectieuses et Immunologie Clinique, Centre Hospitalier Universitaire Henri-Mondor, Assistance Publique-Hôpitaux de Paris (AP-HP), Université Paris-Est Créteil (UPEC), Créteil, France; ^5^Institut Cochin, Inserm U1016, Centre National de la Recherche Scientifique (CNRS) UMR8104, Université Paris-Cité, Paris, France; ^6^Faculté de Médecine and Centre de Recherche ARThrite, Université Laval, Québec, QC, Canada; ^7^Centre de Recherche du Centre Hospitalier Universitaire de Québec, Université Laval, Québec, QC, Canada; ^8^Etablissement Français du Sang Auvergne-Rhône-Alpes, Saint-Etienne, France; ^9^SAINBIOSE, INSERM, U1059, University of Lyon, Saint-Etienne, France; ^10^Division of Infectious Diseases, Queen’s University, Kingston, ON, Canada

**Keywords:** immune activation (IA), extracellular vesicle (EV), people living with HIV (PLWH), microparticles (MPs), immunoregulatory molecules, chronic immune activation

## Abstract

**Introduction:**

People living with HIV (PLWH) now benefit from combined antiviral treatments that durably control viral replication. These antiretroviral treatments decrease mortality and improve quality of life in PLWH, but do not completely control the excessive non-specific activation of the immune system in PLWH. This chronic immune activation is a key element of HIV immunopathology that contributes to the pathophysiology of inflammatory comorbid conditions, such as cardiovascular disorders, cancer and autoimmune diseases. Circulating non-exosomal extracellular vesicles, also known as microparticles (MPs) are detected in these diseases and have been linked to immune activation. The objective of this study was to characterize the MPs present in PLWH and to assess their association with chronic immune activation.

**Methods:**

We performed flow cytometry for the complete phenotypic characterization of MPs from fresh plasma from PLWH and from people without HIV as the control group. The absolute number, size and cellular origin of MPs were evaluated. The immunoregulatory profile was determined by cell origin, for MPs derived from platelets (PMPs), monocytes (MMPs) and T lymphocytes (LMPs).

**Results:**

PLWH had significantly more circulating MPs than controls, for MPs of all sizes originating from T lymphocytes, red blood cells, neutrophils, dendritic cells, B lymphocytes and endothelial cells. PMPs and MMPs were not more numerous in PLWH, but the immunoregulatory phenotypes of these MPs differed between PLWH and controls. These differences in immunoregulatory molecule expression profile were also observed for LMPs. PDL1, ICOSL, CCR5, TGFβ1, MHC classes I and II, TRAIL, CXCR4, OX40, DC-SIGN, CTLA4 and PDL2 were more strongly expressed on the surface of MPs from PLWH than on those from controls.

**Conclusion:**

MPs are an important element in intercellular communication, making it possible to transfer phenotypes and functions to immune cells. The significantly higher numbers of MPs expressing diverse immunomodulatory molecules in PLWH may make a major contribution to the maintenance and/or the development of immune-cell activation in these individuals.

## Introduction

Antiretroviral treatment (ART) controls viral replication in people living with HIV (PLWH), thereby increasing life expectancy, but successful treatment requires high levels of adherence and ART can have multiple adverse effects in long-term use. PLWH on ART have a higher life expectancy due to a decrease in the incidence of opportunistic diseases associated with immune recovery, but they may experience other non-infectious diseases, such as cardiovascular diseases and cancer, which may be favored by excessive non-specific chronic activation of the immune system. This chronic activation contributes an immune aging process, constitutes a significant barrier to the development of immune therapy and is a key factor in the immunopathology of HIV (1).

The extracellular vesicles (EVs) secreted by cells can be classified into several subgroups, including exosomes, apoptotic bodies, and non-exosomal extracellular vesicles, which are also known as microparticles (MPs), microvesicles, or ectosomes (2–4). MPs are generally larger (200-900 nm) than exosomes and are generated by budding of the plasma membrane (5). These vesicles are present in the blood and carry membrane and cytoplasmic elements, such as membrane receptors, cytokines and nucleic acids, from the cell of origin (5–7).

MPs are involved in intercellular communication and can modulate the immune system through interactions with many immune cells (conventional CD4^+^ TL, Tfh, Th17, Treg, monocytes, B lymphocytes, dendritic cells) (8–15). The underlying mechanisms remain incompletely understood, but these interactions may involve immune ligands/receptors present on the surface of the MPs. We recently showed that CD27^+^ and CD70^+^ MPs can transfer these receptors to CD4^+^ TLs, thereby increasing activation and lymphoproliferation (11).

MPs are also linked to the pathogenesis of viral infections, through roles in viral dissemination, inflammation, and modulation of the immune system (12, 16, 17). EVs have been shown to play an important role in the diffusion of HIV infection via the transfer of coreceptors, notably CCR5 and CXCR4 (18, 19). They may also reduce HIV-associated humoral responses by inhibiting the *in vitro* production of immunoglobulins G and A by memory B cells (20). However, almost all studies on EVs and HIV have focused exclusively on the total population of EVs (21–23). As a result, it is not possible to draw any firm conclusions on the role of a specific subset of EVs, such as MPs, in HIV infection and the modulation of immune responses. Nevertheless, we know that the concentration of circulating MPs increases in PLWH (20, 24–26) and that the MP phenotype may be altered in PLWH, which may have an even greater impact on the activation state of immune cells and the regulation of their response.

We used flow cytometry for extensive characterization of the phenotypes of MPs from fresh plasma obtained from PLWH, comparing the results for these individuals with those for people without HIV. We determined the absolute number, size, and cellular origin of MPs. We also characterized the proteins expressed on the membrane of these MPs with a panel of cellular activation and immune checkpoint markers potentially involved in chronic immune cell activation. The immunoregulatory profile of MPs by cell origin was also specified for those with the strongest immunoregulatory potential: MPs derived from platelets (PMPs), monocytes (MMPs) and T lymphocytes (LMPs) (8, 9, 15, 27, 28).

## Materials and methods

### Study population

We used fresh blood samples from patients followed in the infectious diseases and clinical immunology department of Henri-Mondor University Hospital (Creteil, France). The patients included had at least five years of follow-up for HIV infection. They were on active antiretroviral therapy, had an undetectable viral load (< 50 copies/mL) and had a CD4/CD8 ratio greater than 0.5. Demographics, clinical and treatment characteristics, and virological outcomes were collected. Healthy blood donors were enrolled as a control group for comparison with the PLWH, with whom they were matched for sex, ethnicity, and body mass index (BMI) (**Supplementary Tables S1**, **S2**). Blood samples from the control group were provided by the French national blood bank (*Etablissement Français du Sang*, EFS). None of the controls had had an infection (bacterial, viral, fungal, yeast) or had been vaccinated in the 30 days preceding inclusion. The study was approved by the local Ethics Committee, and all participants gave written informed consent.

### MP-enriched EV preparation

MP-enriched EVs were isolated as previously described (9, 10, 29), by differential centrifugation at an initial speed of 3,000 x *g* at 4°C for 10 minutes. The supernatant was then centrifuged at 13,000 x *g* at 4°C for 10 minutes for the preparation of a platelet-free supernatant. MP-enriched EVs were concentrated by centrifuging the platelet-free supernatant for 1 hour at 100,000 x *g* at 4°C. MP-enriched EVs were resuspended in filtered PBS (filtration through a 0.1 μm pore-size PES membrane) and MPs were characterized by flow cytometry detection.

### MP phenotyping

MPs were labeled as previously described (10, 11, 29), with fluorochrome-conjugated monoclonal antibodies. MPs were labeled with five different panels from the antibodies described in **Supplemental Table S3**. Fluorescence was assessed with a 20-parameter LSR Fortessa flow cytometer with a small-particle option (BD Biosciences) based on photomultiplier (PMT)-coupled forward scatter (FSC) detection. This mode of detection was used to ensure the optimal detection of MPs with diameters of 200 to 900 nm. The performance of the flow cytometer was checked before each assay. Megamix-Plus FSC and SSC beads (BioCytex, Marseille, France) of known dimensions (with mean diameters of 200 nm, 500 nm and 900 nm) were used for the standardization of FSC-PMT parameters and definition of the MP gate. MPs were acquired at low speed and quantified in Trucount tubes (BD Biosciences).

### Flow cytometry analysis

Flow cytometry data were analyzed with FlowJo software (v.10.7.1, Ashland, OR).

### Statistical analysis

All analyses were performed with Prism 6.07 software (GraphPad Software, La Jolla, CA). Only significant differences between groups (*P*<0.05) are indicated on the data plots.

## Results

### Characterization of MPs in PLWH

**Figure 1:**
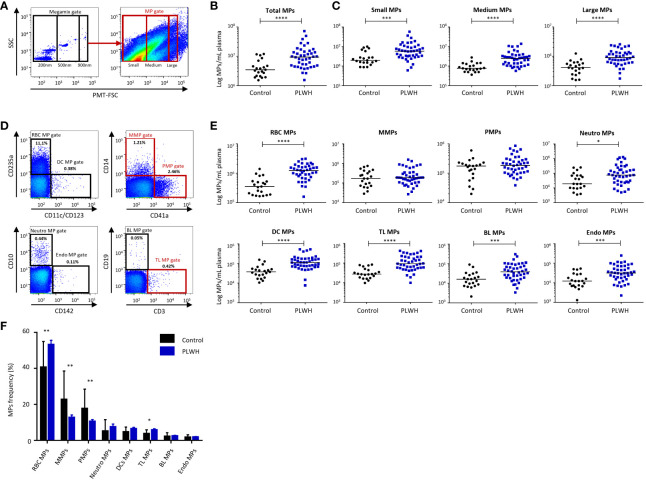
Distribution of MPs by size and cellular origin: comparison of PLWH and controls. **(A)** Example of the gating strategy used for MP phenotyping by flow cytometry for the control group (*n* = 21) and PLWH (*n* = 42) (21 experiments with 2 patients and 1 control per experiment). On the left, a dot plot showing the settings based on fluorescent beads for the differentiation of three particle sizes: 200, 500 and 900 nm in diameter. On the right, dot plot for MP acquisition on a Fortessa flow cytometer for one representative PLWH. **(B)** Total MPs and **(C)** MPs of various sizes were quantified by flow cytometry and their concentrations were determined with Trucount tubes. **(D)** Example of the gating strategy used for the cellular origin phenotyping of MPs by flow cytometry for the control group (*n* = 21) and PLWH (*n* = 42) (21 experiments with 2 patients and 1 control per experiment). RBC MPs, MMPs, PMPs, Neutro MPs, DC MPs, TL MPs, BL MPs and Endo MPs were acquired in the MP gate. The indicated percentages represent the cell subpopulations of the MPs out of the total population. Representative FACS plots for each cellular subtype of MPs are shown. **(E)** Each cellular subtype of MPs was quantified by flow cytometry and MP concentrations were determined with Trucount tubes. The cellular markers are arranged in descending order of the number of MPs/mL expressing them in plasma. Horizontal bars indicate the median values. **(F)** The frequencies of CD235a^+^ RBC MP, CD14^+^ MMPs, CD41a^+^ PMPs, CD10^+^ Neutro MPs, CD11c^+^, CD123^+^ DC MPs, CD3^+^ TL MPs, CD19^+^ BL MPs and CD142^+^ Endo MPs were determined by flow cytometry for the control group (*n* = 21) and PLWH (*n* = 42) (21 experiments, with 2 patients and 1 control per experiment). The percentages were reported on the totality of MPs of cellular origin studied. *P* values (*P*<0.05 considered significant) were obtained in Mann-Whitney and *post hoc* tests. **** *P*<0.0001, *** *P*<0.001, ** P<0.01, * *P*<0.05.

We compared the profiles of MPs from fresh plasma between PLWH (*n* = 42) and controls (*n* = 21) (**Figure 1**). The MP gate extended from just below the 200 nm bead signal up to the 900 nm bead signal on the side scatter (SSC) and photomultiplier (PMT)-forward scatter (FSC) dot plot (**Figure 1A**). The number and size distribution of MPs were determined by considering three size classes: small (< 300 nm), medium (300-500 nm), and large (> 500 nm) MPs (**Figures 1A–C**).

PLWH had significantly more circulating MPs than the controls (13.1x10^6^ ± 2.1x10^6^ vs. 4.7x10^6^ ± 0.7x10^6^ MPs/ml plasma, *P*<0.0001) (**Figure 1B**). This large increase in the number of MPs concerned MPs of all sizes: small MPs, 8.8x10^6^ ± 1.6x10^6^ vs. 3.2x10^6^ ± 0.6x10^6^ MPs/ml plasma (*P*=0.0003); medium-sized MPs, 3.2x10^6^ ± 0.5x10^6^ vs. 1.0x10^6^ ± 0.1x10^6^ MPs/ml plasma (*P*<0.0001); large MPs, 1.1x10^6^ ± 0.1x10^6^ vs. 0.5x10^6^ ± 0.1x10^6^ MPs/ml plasma (*P*<0.0001) (**Figure 1C**).

### Cellular origin of MPs in PLWH

We analyzed subpopulations of MPs defined on the basis of their cellular origin, as determined by evaluating the expression of surface proteins specific to red blood cells (CD235a^+^ RBC MPs), platelets (CD41a^+^ PMPs), monocytes (CD14^+^ MMPs), neutrophils (CD10^+^ Neutro MPs), dendritic cells (CD11c^+^, CD123^+^ DC MPs), T lymphocytes (CD3^+^ TL MPs), B lymphocytes (CD19^+^ BL MPs) and endothelial cells (CD142^+^ Endo MPs) (**Figures 1D–F**).

The concentrations of MPs of all cell origins other than platelets or monocytes (PMPs or MMPs) were significantly higher in PLWH than in controls: for RBC MPs, 1.3x10^6^ ± 0.1x10^6^ vs. 0.4x10^6^ ± 0.1x10^6^ MPs/ml (*P*<0.0001); for DC MPs, 14.5x10^4^ ± 1.9x10^4^ vs. 4.7x10^4^ ± 0.8x10^4^ MPs/ml (*P*<0.0001); for Neutro MPs, 20.6x10^4^ ± 4.9x10^4^ vs. 5.8x10^4^ ± 1.6x10^4^ MPs/ml (*P*=0.0101); for TL MPs, 13.6x10^4^ ± 1.7x10^4^ vs. 3.6x10^4^ ± 0.5x10^4^ MPs/ml (*P*<0.0001); for BL MPs, 5.8x10^4^ ± 0.9x10^4^ vs. 2.2x10^4^ ± 0.5x10^4^ MPs/ml (*P*=0.0007); for Endo MPs, 4.7x10^4^ ± 0.7x10^4^ vs. 1.8x10^4^ ± 0.3x10^4^ MPs/ml (*P*=0.0008) (**Figure 1E**).

We also compared the frequencies of each subtype of MPs based on cell origin between PLWH and controls (**Figure 1F**). RBC MPs, PMPs and MMPs were each present at frequencies of more than 10% and together accounted for about 80% of the MPs studied in blood (**Figure 1F**). The frequencies of RBC MPs and TL MPs were significantly higher in PLWH than in controls (RBC MPs, 53.2% ± 2.4% vs. 40.7% ± 3.1%, *P*=0.004); TL MPs, 5.9% ± 0.6% vs. 3.8% ± 0.5%, *P*=0.034). However, the frequencies of MMPs and PMPs were significantly lower in PLWH than in controls (MMPs, 12.8% ± 1.4% vs. 22.8% ± 3.4%, *P*=0.008; PMPs, 10.6% ± 1.0% vs. 17.7% ± 2.3%, *P*=0.006) (**Figure 1F**). We found no correlation between cellular and MP phenotypes, either in PLWH or in the control group (data not shown).

### Immunoregulatory profile of total MPs in PLWH

**Figure 2:**
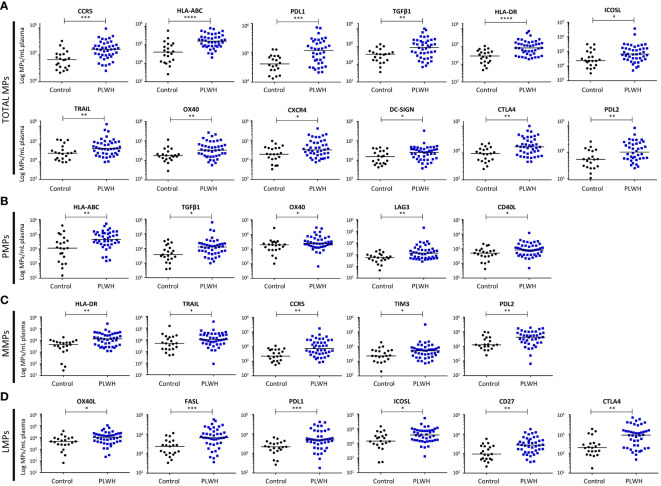
Immunoregulatory molecule phenotypes of MPs from PLWH and the control group. The expression of immunoregulatory markers on the surface of total MPs **(A)**, CD41a^+^ PMPs **(B)**, CD14^+^ MMPs **(C)** and CD3^+^ LMPs **(D)** was quantified by flow cytometry on fresh plasma samples from the control group (*n* = 21) and PLWH (*n* = 42) (21 experiments, with 2 patients and 1 control per experiment). The concentrations of MPs expressing each of the markers were determined with Trucount tubes. The immunoregulatory markers are arranged in descending order of the number of MPs/mL of plasma expressing them. Horizontal bars indicate the median values. *P* values (*P*<0.05 considered significant) were obtained in Mann-Whitney and *post hoc* tests. **** *P*<0.0001, *** *P*<0.001, ** *P*<0.01, * *P*<0.05.

We characterized the immunoregulatory profile of total MPs from PLWH and controls. We performed flow cytometry to assess the membrane expression of 23 cellular activation and immune checkpoint markers potentially involved in chronic immune activation (PD1, ICOSL, OX40L, CD40L, LAG3, TGF-β1, PDL1, PDL2, HLA-ABC, CD86, DC-SIGN, CLEC2, CXCR4, CCR5, CD40, OX40, FASL, TIM3, HLA-DR, TRAIL, CTLA4, CD27 and CD39) (**Supplementary Figures 1**, **2**, **Figure 2**).

We identified 12 immunoregulatory molecules among the markers tested that were more strongly expressed by the MPs of PLWH than by those of controls (**Figure 2A**). The markers on total MPs that did not differ significantly between groups are presented in **Supplementary Figure 2**.

For ligands of PD1, the differences between PLWH and the control group were as follows: for PDL1^+^ MPs, 1.7x10^5^ ± 0.3x10^5^ vs. 0.5x10^5^ ± 0.1x10^5^ MPs/ml, (*P*=0.0006), and for PDL2^+^ MPs, 1.6x10^4^ ± 0.3x10^4^ vs. 0.7x10^4^ ± 0.1x10^4^ MPs/ml (*P*=0.003).

For MHC I and II molecules, the differences in expression on MPs between PLWH and controls were as follows: for HLA-ABC^+^ MPs, 2.2x10^5^ ± 0.3x10^5^ vs. 0.8x10^5^ ± 0.3x10^5^ MPs/ml (*P*<0.0001), and for HLA-DR^+^ MPs, 1.3x10^5^ ± 0.2x10^5^ vs. 0.4x10^5^ ± 0.1x10^5^ MPs/ml (*P*<0.0001).

For the costimulation molecules ICOSL and OX40, the differences between PLWH and controls were as follows: for ICOSL^+^ MPs, 2.2x10^5^ ± 0.9x10^5^ vs. 0.7x10^5^ ± 0.2x10^5^ MPs/ml (*P*=0.016), and for OX40^+^ MPs, 5.5x10^4^ ± 0.1x10^4^ vs. 2.4x10^4^ ± 0.1x10^4^ MPs/ml (*P*=0.008).

For the other costimulation molecules considered, TGF-β1, TRAIL and CTLA4, the differences between PLWH controls were as follows: for TGF-β1^+^ MPs, 1.6x10^5^ ± 0.3x10^5^ vs. 0.4x10^5^ ± 0.1x10^5^ MPs/ml (*P*=0.002); for TRAIL^+^ MPs, 1.3x10^5^ ± 0.3x10^5^ vs. 0.5x10^5^ ± 0.1x10^5^ MPs/ml (*P*=0.0241), and for CTLA4^+^ MPs, 2.3x10^4^ ± 0.3x10^4^ vs. 1.0x10^4^ ± 0.1x10^4^ MPs/ml (*P*=0.002).

Chemokine receptors were also more strongly expressed on the MPs of PLWH than on those of controls: for CCR5^+^ MPs, 2.0x10^5^ ± 0.3x10^5^ vs. 0.9x10^5^ ± 0.2x10^5^ MPs/ml plasma (*P*=0.0003), and for CXCR4^+^ MPs, 6.3x10^4^ ± 0.1x10^4^ vs. 2.8x10^4^ ± 0.5x10^4^ MPs/ml plasma (*P*=0.039).

DC-SIGN was also more strongly expressed on the MPs from PLWH than on those of controls: 3.4x10^4^ ± 0.8x10^4^ vs. 1.9x10^4^ ± 0.3x10^4^ MPs/ml plasma (*P*=0.04).

### Immunoregulatory profiles of PMPs, MMPs and LMPs in PLWH

We then evaluated the immunoregulatory profile of MPs according to their cell type of origin, focusing particularly on PMPs, MMPs and LMPs, which are derived from cell subsets known to be involved in immunoregulation (8–11, 13, 14).

#### CD41a^+^ PMPs

MPs from platelets can account for up to 32% of the MPs circulating in the blood of PLWH (**Figure 1F**) and are therefore a potentially important factor in innate and adaptive immunity. The immunoregulatory molecule expression profile of the PMPs of PLWH was characterized by flow cytometry based on 15 surface markers: HLA-ABC, TGFβ1, CXCR4, ICOSL, CLEC2, DC-SIGN, CD40, CD86, OX40, OX40L, CCR5, CD39, CD40L, LAG3, PD1 (**Supplementary Figure 3**, **Figure 2B**). We observed a significant overexpression of five immunoregulatory molecules on the PMPs of PLWH relative to controls: HLA-ABC, 8.6x10^4^ ± 1.6x10^4^ vs. 4.8x10^4^ ± 2.0x10^4^ MPs/ml (*P*=0.004); TGF-β1, 3.6x10^4^ ± 1.5x10^4^ vs. 0.9x10^4^ ± 0.2x10^4^ MPs/ml (*P*=0.014); OX40, 4.7x10^3^ ± 0.9x10^3^ vs. 3.0x10^3^ ± 1.3x10^3^ MPs/ml (*P*=0.04); CD40L, 1.4x10^3^ ± 0.3x10^3^ vs. 0.7x10^3^ ± 0.1x10^3^ MPs/ml (*P*=0.03) and LAG3, 7.4x10^3^ ± 4.8x10^3^ vs. 0.7x10^3^ ± 0.1x10^3^ MPs/ml (*P*=0.002) (**Figure 2B**). The markers on PMPs that did not differ significantly between groups are presented in **Supplementary Figure 3**.

#### CD14^+^ MMPs

MPs derived from monocytes can account for up to 38% of the circulating MPs in the blood of PLWH (**Figure 1F**) and may have effects on inflammation, oxidative stress and cell apoptosis. We performed flow cytometry to characterize the immunoregulatory molecule profiles of MMPs for 16 markers: PDL1, HLA-DR, TRAIL, OX40L, ICOSL, FASL, CD86, CCR5, TIM3, CXCR4, CD39, PDL2, TGFβ1, LAG3, CD40L, PD1 (**Supplementary Figure 4**, **Figure 2C**). Five immunoregulatory molecules were significantly overexpressed on MMPs from PLWH relative to MMPs from the control group: HLA-DR, 2.5x10^4^ ± 0.7x10^4^ vs. 0.5x10^4^ ± 0.1x10^4^ MPs/ml (*P*=0.001); TRAIL, 2.5x10^4^ ± 0.8x10^4^ vs. 1.5x10^4^ ± 0.7x10^4^ MPs/ml (*P*=0.022); CCR5, 1.8x10^4^ ± 0.4x10^4^ vs. 0.4x10^4^ ± 0.1x10^4^ MPs/ml (*P*=0.001); TIM3, 1.4x10^4^ ± 0.8x10^4^ vs. 0.4x10^4^ ± 0.1x10^4^ MPs/ml (*P*=0.044), and PDL2, 5.8x10^3^ ± 0.8x10^3^ vs. 2.5x10^3^ ± 0.6x10^3^ MPs/ml (*P*=0.005) (**Figure 2C**). The markers on MMPs that did not differ significantly between groups are presented in **Supplementary Figure 4**.

#### CD3^+^ LMPs

MPs derived from lymphocytes can account for up to 18% of the MPs circulating in the blood of PLWH (**Figure 1F**). The mean frequency of LMPs among total circulating MPs is only 6%, but even small numbers of LMPs can exert immunomodulatory effects, particularly on lymphocytes. The surface immunoregulatory molecule profile of the LMPs was characterized by flow cytometry with 12 markers: OX40L, CD39, ICOSL, FASL, PDL1, CD27, LAG3, TGFβ1, TRAIL, CD40L, PD1 and CTLA-4 (**Supplementary Figure 5**, **Figure 2D**). We found that six immunomodulatory molecules were more strongly expressed on LMPs from PLWH than on LMPs from controls: OX40L, 1.6x10^4^ ± 0.3x10^4^ vs. 0.7x10^4^ ± 0.2x10^4^ MPs/ml (*P*=0.01); ICOSL, 6.2x10^3^ ± 1.3x10^3^ vs. 2.9x10^3^ ± 0.7x10^3^ MPs/ml (*P*=0.01); FASL, 11.3x10^3^ ± 1.9x10^3^ vs. 3.0x10^3^ ± 0.6x10^3^ MPs/ml (*P*=0.0002); PDL1, 8.8x10^3^ ± 1.6x10^3^ vs. 2.5x10^3^ ± 0.7x10^3^ MPs/ml (*P*=0.0009); CD27, 3.9x10^3^ ± 0.6x10^3^ vs. 1.5x10^3^ ± 0.3x10^3^ MPs/ml (*P*=0.001), and CTLA4, 14.7x10^2^ ± 2.6x10^2^ vs. 4.0x10^2^ ± 1.1x10^2^ MPs/ml (*P*=0.001). (**Figure 2D**). The markers on LMPs that did not differ significantly between groups are presented in **Supplementary Figure 5**.

Finally, despite the identical activation profiles of PLWH and control MPs (**Supplementary Figure 6**), the number of immunomodulatory MPs was greater in PLWH than controls.

## Discussion

We found that PLWH on ART had significantly larger numbers of MPs in their plasma than controls. This increase in MP numbers, which points to cellular activation, is potentially important because MPs can have various effects, depending on their phenotype and numbers (9–11). Many factors, including aging, can influence the production of MPs even in people without HIV (30). However, we observed no correlation between age and the number of MPs in either PLWH or controls (data not shown).

We found that the increase in the number of MPs in PLWH was not dependent on MP size, with all size classes, including the largest (900 nm), affected. This finding is important, because the largest MPs may contain organelles, such as mitochondria, which may participate in the production of anti-cardiolipin antibodies (12). These antibodies play an important role in anti-phospholipid antibody syndrome and are responsible for the development of a state of thrombophilia. Other MPs may contain proteasomes, which can contribute to antigen presentation through class I MHC (14).

Moreover, the phenotypic characteristics of MPs are key to identification of the cellular targets of MPs in the immune system. Indeed, the interactions of MPs with immune cells differ according to the cellular origin of the MPs and their immunoregulatory phenotype (9, 11). The interaction between MPs and immune cells is not random and involves molecules present on the surface of MPs (11). This interaction can result in a transfer of molecules from the MP to the immune cell, with immunomodulatory consequences. We show here that the increase in MP numbers in PLWH concerns not only MPs from red blood cells and endothelial cells, but also other MPs derived from immune system cells, such as neutrophils, dendritic cells and T and B lymphocytes. This increase cannot be explained by an increase in the number of cells because we have never observed any correlation between cellular expression and MPs, in either controls (9) or PLWH (data not shown). Furthermore, we have recently shown that the role of MPs depends on their quantity (9). We have shown that LMPs, even if present at only very low levels, can facilitate both cellular and humoral responses. These findings support the possible maintenance of an inflammatory endothelial environment, and immune activation through specific interactions of MPs with the immune system (11).

Only MPs derived from monocytes and platelets were present in similar numbers in both the PLWH and control groups. However, the phenotype of these MPs differed between PLWH and controls. Indeed, the analysis of immunomodulatory molecule expression on the surface of PMPs indicated that class I MHC molecules (HLA-ABC), TGFβ1, OX40 (CD134), LAG3 (CD223) and CD40L (CD154) were more strongly expressed on the PMPs of PLWH than on those of controls. These data confirm the state of chronic platelet activation described in PLWH patients (31) and also clearly suggest a role for these MPs in immune activation (9).

Differences in the immunomodulatory molecules expressed on the surface were also found for MMPs, but for the molecules concerned were different, with an overexpression of molecules capable of modulating immune activation, such as class II MHC (HLA-DR), TRAIL (CD253), TIM3 (CD366) and PDL2 (CD273) in PLWH relative to controls. The MMPs of PLWH also had higher levels of CCR5 expression on their surface. MMPs could, thus, play a key role in the spread of the virus and the maintenance of virus reservoirs (14, 18).

Similarly, LMPs from PLWH had significantly higher levels of expression for OX40L (CD252), ICOSL (CD275), FASL (CD178), PDL1 (CD274), CTLA4 (CD152) and CD27 than those from controls. These findings clearly suggest that these MPs could play a role in the transfer or maintenance of T-lymphocyte immune activation, as reported in our previous studies (11). Indeed, CD27^+^ MPs bind only to cells that already express CD27. This binding results partly from the co-expression of CD70 by these CD27^+^ MPs (11). These data testify to the ability of MPs to reprogram cells via marker transfer, thereby maintaining an immune phenotype.

We also observed much stronger DC-SIGN and CXCR4 expression on MPs from PLWH than on those from controls. The cellular origin of the MPs overexpressing these two markers remains unclear, but we suspect that they are derived from dendritic cells and/or B lymphocytes and can participate in the maintenance and dissemination of the virus. Furthermore, it has been known since the early 2000s that CXCR4 and CCR5 are present on MPs and can transfer a capacity for infection to cells lacking these receptors (18, 19). This ability of MPs to transfer markers suggests that DC-SIGN and CXCR4 MPs may be associated with the maintenance of a viral load in reservoirs.

Despite the careful application of selection criteria during the recruitment of participants (HIV infection, patient on treatment with subsequent immune reconstitution), phenotyping results for MPs were highly heterogeneous, particularly for PDL1, TGFβ1 and CTLA4. This variability suggests that comorbid conditions in these patients may drive the characteristic expression of immunomodulatory markers on the surface of MPs. Indeed, these MPs with particular phenotypes may be associated with comorbid conditions and chronic activation of the immune system.

Other chronic co-infections and the resulting activation of the immune system may also explain these higher levels of MPs, but we found no link between the number of MPs and chronic co-infection with HBV or HCV (data not shown).

Finally, we hypothesize that antiviral treatments may direct the production of MPs. We found no correlation between the frequency or phenotype of MPs and ART treatment (data not shown), but we believe that this lack of correlation may result from treatment heterogeneity, which might also account for the occurrence of comorbidities as a function of the MPs induced. The only information available to support this hypothesis comes from studies on the effects on RBCs of ART. Indeed, Peltenburg et al. have shown that abacavir treatment is associated with an increase in cardiovascular risk, whereas no such increase in risk is observed for tenofovir or didanosine (32, 33). However, it remains unclear whether this risk is associated with RBC-MPs. There are also indirect links, such as the impact on cholesterol and, hence, on MP survival (34), and the effect of nucleoside reverse transcriptase inhibitors on RBC (35). Dolutegravir is also directly associated with the apoptosis of RBCs (36) or other cell types (37), inflammation (38), and platelet activation correlated with an increase in the risk of cardiometabolic comorbidities (39). This last point links platelet activation and the production of PMPs (12).

The data obtained here are therefore of potential value for the development of new treatments for reducing the impact of these MPs, whether or not they are linked to infection. Indeed, new therapeutic or vaccination strategies based on the specific transfer of functions from MPs could be envisaged (11). However, as we recently showed with CD27^+^ MPs, it is important to take the contribution of immunoregulatory MPs into account, particularly in patients treated with immunotherapies based on monoclonal antibodies (11).

## Data availability statement

The original contributions presented in the study are included in the article/**Supplementary Material**. Further inquiries can be directed to the corresponding author.

## Ethics statement

The studies involving humans were approved by Comite de Protection des Personnes - Ile-de-France IX. The studies were conducted in accordance with the local legislation and institutional requirements. The participants provided their written informed consent to participate in this study.

## Author contributions

DN-L: Formal analysis, Investigation, Writing – original draft. MT: Writing – review & editing. RB: Writing – review & editing. SM: Writing – review & editing, Investigation. PV: Writing – review & editing, Investigation. JL: Writing – review & editing, Investigation. AD: Writing – review & editing. MA: Writing – review & editing. EB: Writing – review & editing. FC: Writing – review & editing. HH-C: Writing – review & editing. SP: Writing – review & editing. J-DL: Writing – review & editing. FP: Writing – review & editing. SG: Writing – review & editing. BV: Conceptualization, Supervision, Validation, Writing – original draft.
