# Supplementary material for: Immunoregulatory molecule expression on extracellular microvesicles in people living with HIV
Source: Front Immunol. 2024 Mar 4;15:1354065. doi: 10.3389/fimmu.2024.1354065 (PMC10944887; doi:10.3389/fimmu.2024.1354065)
Supplement: Supplementary file 1 [file DataSheet_1.docx]

**Immunoregulatory molecule expression on extracellular microvesicles in people living with HIV**

Deborah Neyrinck-Leglantier, Marie Tamagne, Raida Ben Rayana, Sounagya Many, Paul Vingert, Julie LeGagneux, Adèle Silane Delorme, Muriel Andrieu, Eric Boilard, Fabrice Cognasse, Hind Hamzeh-Cognasse, Santiago Perez-Patrigeon, Jean-Daniel Lelievre, France Pirenne, Sébastien Gallien, and Benoît Vingert.

**Supplemental Data**

**Supplemental Table 1.** Individual characteristics of PLWH

**Supplemental Table 2.** Characteristics of the study population

**Supplemental Table 3.** Fluorescent antibodies for MPs and whole-blood phenotyping

**Supplemental Figure 1.** Gating strategy for immunoregulatory molecules expressed by MPs.

**Supplemental Figure 2.** Immunoregulatory molecule phenotype of total MPs from PLWH.

**Supplemental Figure 3.** Immunoregulatory molecule phenotype of PMPs from PLWH.

**Supplemental Figure 4.** Immunoregulatory molecule phenotype of MMPs from PLWH.

**Supplemental Figure 5.** Immunoregulatory molecule phenotype of TL MPs from PLWH.

**Supplemental Figure 6.** Frequency of each immunoregulatory marker of MPs

**
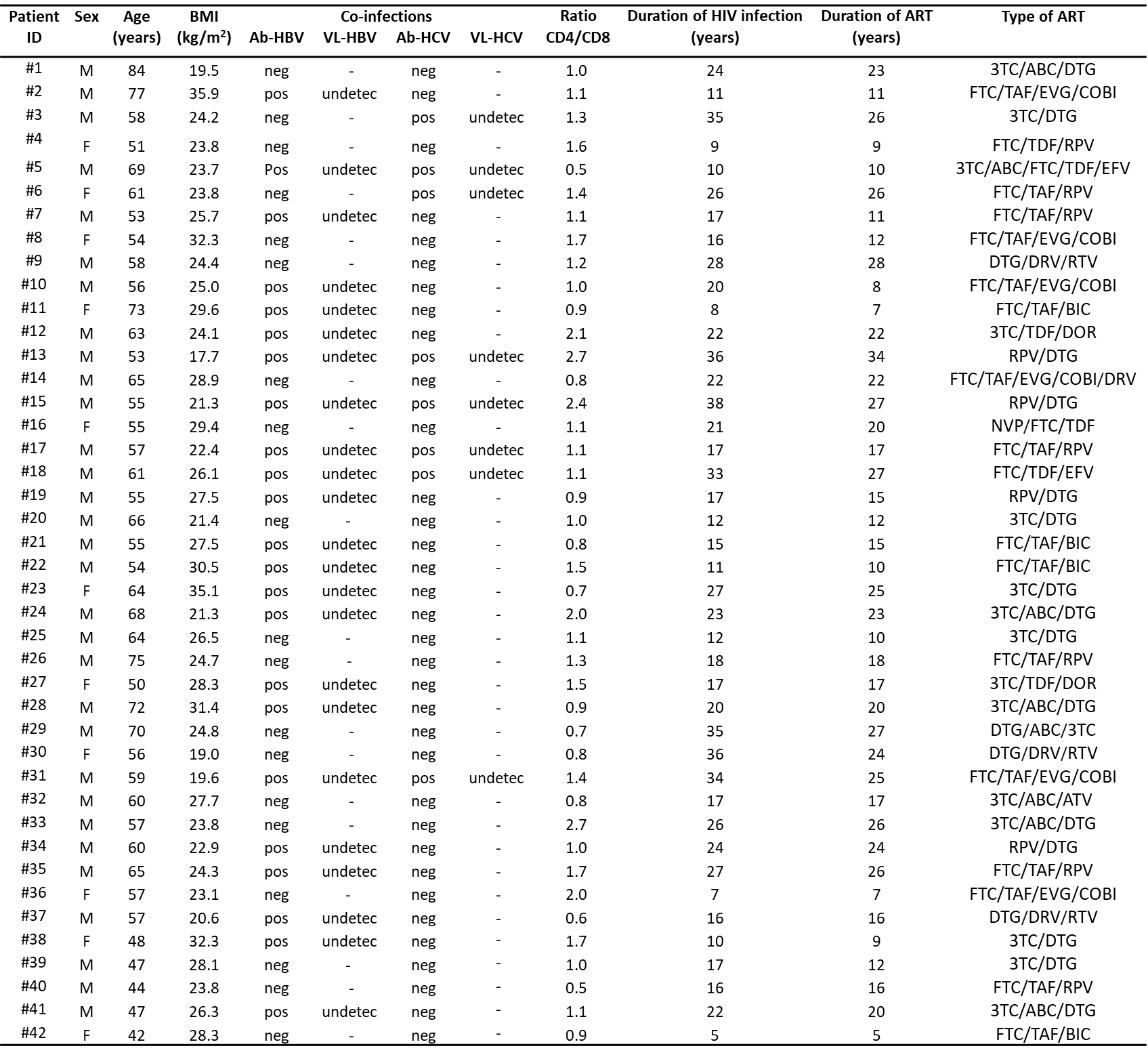
Supplemental Table 1. Individual characteristics of PLWH**

Abbreviations: ABC abacavir, ATV atazanavir, BIC bictegravir, COBI cobicistat, DRV darunavir, DOR doravirine, DTG dolutegravir, EFV efavirenz, EVG elvitegravir, FTC emtricitabine, NVP nevirapine, RPV rilpivirine, RTV ritonavir, TAF tenofovir alafenamide, TDF tenofovir disoproxil fumarate, 3TC lamivudine

**Supplemental Table 2. Characteristics of the study population**

| **Participant characteristics** | **Controls**  **(*n*=21)** | **PLWH**  **(*n*=42)** |
| --- | --- | --- |
| Demographics  Male [*n* (%)]  Age (y) (median [min; max])  BMI (kg/m^2^) (median [min; max]) | 13 (62)  35 [22 ; 64]  25 [19 ; 33] | 31 (74)  58 [42 ; 84]  25 [18 ; 36] |
| Viral-immunological  HIV viral load undetectable [*n* (%)]  CD4^+^ T-cell count (cells/µL) (median [min; max])  Nadir CD4^+^ T-cell count (cells/µL) (median [min;max])  CD8^+^ T-cell count (cells/µL) (median [min; max])  Duration of HIV infection (y) (median [min; max])  Duration of ART (y) (median [min; max]) | NA  ND  NA  ND  NA  NA | 41 (98)  657 [257 ; 1261]  191 [5 ; 658]  594 [176 ; 1144]  19 [5 ; 38]  17 [5 ; 34] |

Abbreviations: ART antiretroviral therapy, PLWH people living with HIV, NS, non-significant (*P*>0.05); BMI, body mass index; NA, not available; ND:  not done

**Supplemental Table 3. Fluorescent antibodies for MP phenotyping**

| **Antibody** |  | **Clone** |  | **Fluorochrome** |  | **Supplier** |
| --- | --- | --- | --- | --- | --- | --- |
| CD10 |  | Hl10a |  | PE-Cy7 |  | BD Biosciences ^$^ |
| CD11c |  | B-ly6 |  | APC |  |  |
| CD14 |  | M5E2 |  | BV650 |  |  |
| CD142 |  | HTF-1 |  | PE |  |  |
| CD16 |  | 3G8 |  | FITC |  |  |
| CD19 |  | HIB19 |  | AF700 |  |  |
| CD3 |  | UCHT1 |  | BUV737 |  |  |
| CD41a |  | HIP8 |  | APC-H7 |  |  |
| CD235a |  | GA-R2 |  | PE-Cy5 |  |  |
| CD154/CD40L |  | TRAP1 |  | PE-CF594 |  |  |
| CD252/OX40L |  | Ik-1 |  | BV421 |  |  |
| CD279/PD1 |  | EH12.1 |  | PE |  |  |
| CD184/CXCR4 |  | 12G5 |  | PE-CF594 |  |  |
| CD195/CCR5 |  | 2D7/CCR5 |  | FITC/AF488 |  |  |
| CD209/DC-SIGN |  | DCN46 |  | APC |  |  |
| CD86/B7.2 |  | 2331 (FUN-1) |  | PerCP-Cy5.5 |  |  |
| CD178/FASL |  | NOK-1 |  | PE |  |  |
| CD253/TRAIL |  | RIK-2 |  | BV421 |  |  |
| CD273/PDL2 |  | MIH18 |  | APC |  |  |
| CD274/PDL1 |  | MIH1 |  | PE-Cy7 |  |  |
| HLA-DR |  | G46-6 |  | APC-H7 |  |  |
| CD152/CTLA-4 |  | BNI3 |  | APC |  |  |
| CD27 |  | L128 |  | BV510 |  |  |
| CD123 |  | 6H6 |  | BV421 |  | BioLegend ^§^ |
| CD223/LAG3 |  | 11C3C65 |  | PerCP-Cy5.5 |  |  |
| CD275/ICOSL |  | 2D3 |  | APC |  |  |
| CD39 |  | TU66 |  | BV510 |  |  |
| TGF-β1 |  | S20006A |  | PE-Cy7 |  |  |
| CD134/OX40 |  | ACT35 |  | BV421 |  |  |
| CD40 |  | 5C3 |  | PE-Cy7 |  |  |
| CLEC2/CLEC1B |  | AYP1 |  | PE |  |  |
| HLA-ABC |  | W6/32 |  | BV510 |  |  |
| CD366/TIM3 |  | F38/2E2 |  | BV510 |  |  |

^$^ BD Biosciences, San Jose, CA

^§^ BioLegend, San Diego, CA

**
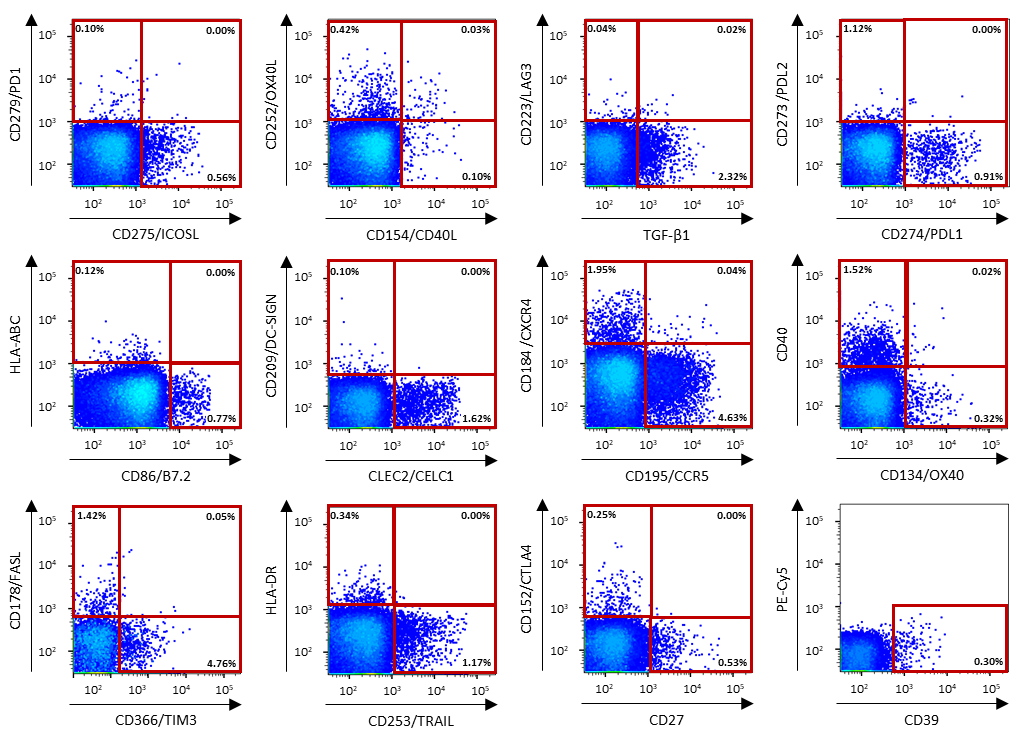
Supplemental Figure 1. Gating strategy for the immunoregulatory molecules expressed by MPs.** Example of the gating strategy used for MP phenotyping by flow cytometry. Fluorescence was assessed on an LSR Fortessa flow cytometer, and the flow cytometry data were analyzed with FlowJo software.

**
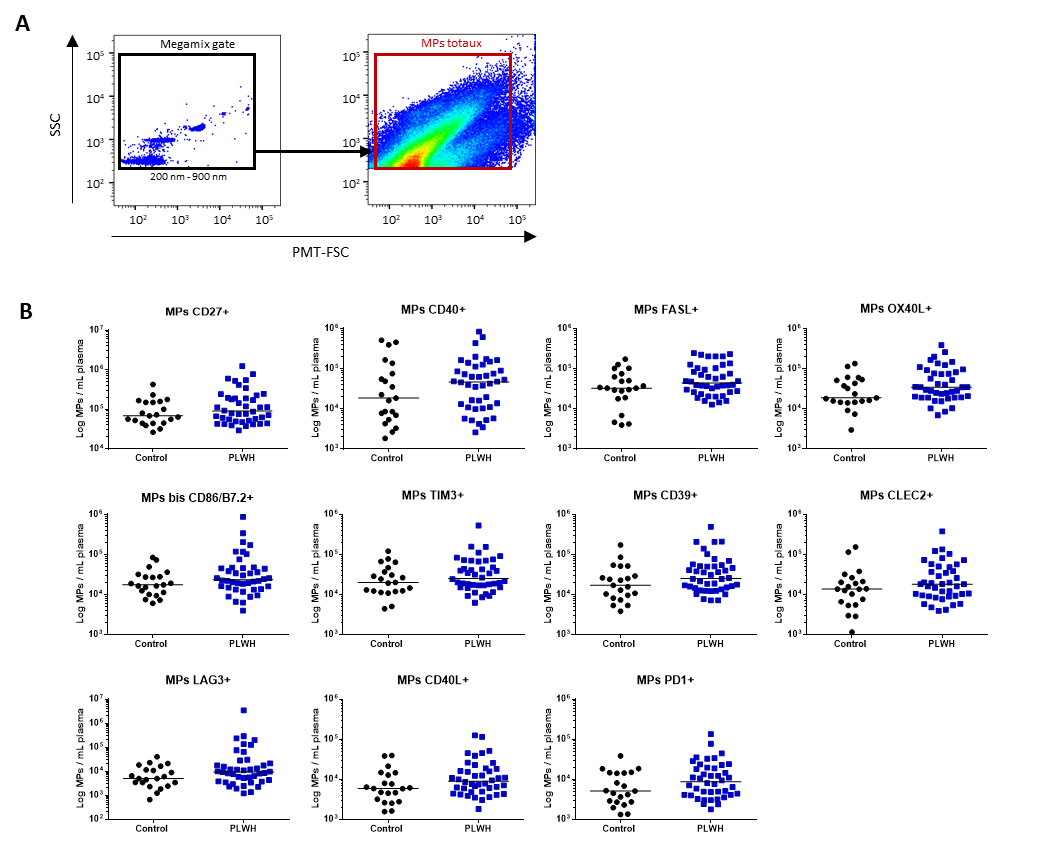
Supplemental Figure 2. Immunoregulatory molecule phenotypes of total MPs from PLWH versus controls. (A)** Example of the gating strategy used in the phenotyping of total MPs by flow cytometry for the control group (*n* = 21) and PLWH (*n* = 42) (21 experiments, with 2 patients and 1 control per experiment). The dot plot on the left shows the settings based on fluorescent beads for the differentiation of three particle sizes: 200, 500 and 900 nm in diameter. The dot plot on the right shows the MP gate used for acquisition on a Fortessa flow cytometer for one representative PLWH. Flow cytometry data were analyzed with FlowJo software. **(B)** The surface expression of immunoregulatory markers on total MPs was quantified by flow cytometry on fresh plasma samples and the concentrations of the MPs expressing each of the markers were determined with Trucount tubes. The immunoregulatory markers are arranged in descending order of the number of MPs/mL of plasma expressing the marker concerned. Horizontal bars indicate the median values. *P* values were obtained in Mann-Whitney tests and post hoc tests.

**
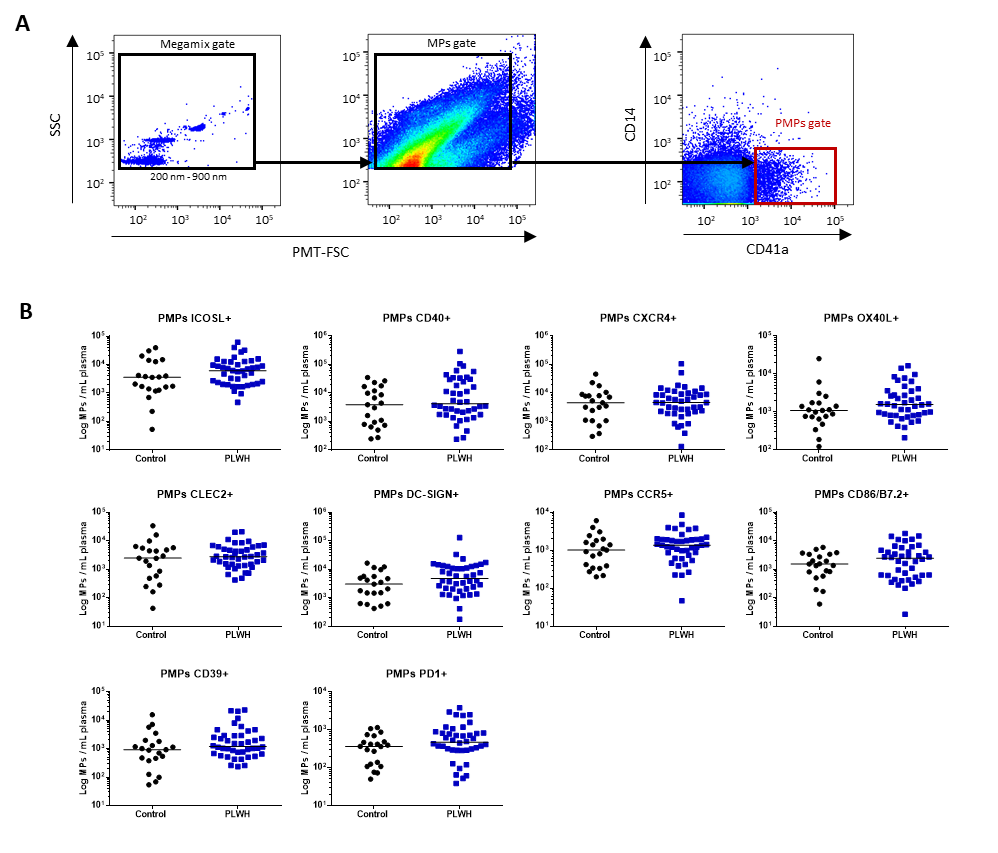
Supplemental Figure** **3. Immunoregulatory molecule phenotype of PMPs from PLWH versus controls. (A)** Example of the gating strategy used in the phenotyping of CD41a^+^ PMPs by flow cytometry for the control group (*n* = 21) and PLWH (*n* = 42) (21 experiments, with 2 patients and 1 control per experiment). The dot plot on the left shows the settings based on fluorescent beads for the differentiation of three particle sizes: 200, 500 and 900 nm in diameter. A dot plot for MPs is shown in the middle, and another for CD41a^+^ PMPs is shown on the right, for acquisition on a Fortessa flow cytometer for one representative PLWH. Flow cytometry data were analyzed with FlowJo software. **(B)** The surface expression of immunoregulatory markers on PMPs was quantified by flow cytometry on fresh plasma samples and the concentrations of PMPs expressing the various markers were determined with Trucount tubes. The immunoregulatory markers are arranged in descending order of the number of MPs/mL of plasma expressing the marker concerned. Horizontal bars indicate the median values. *P* values were obtained in Mann-Whitney tests and post hoc tests.

**
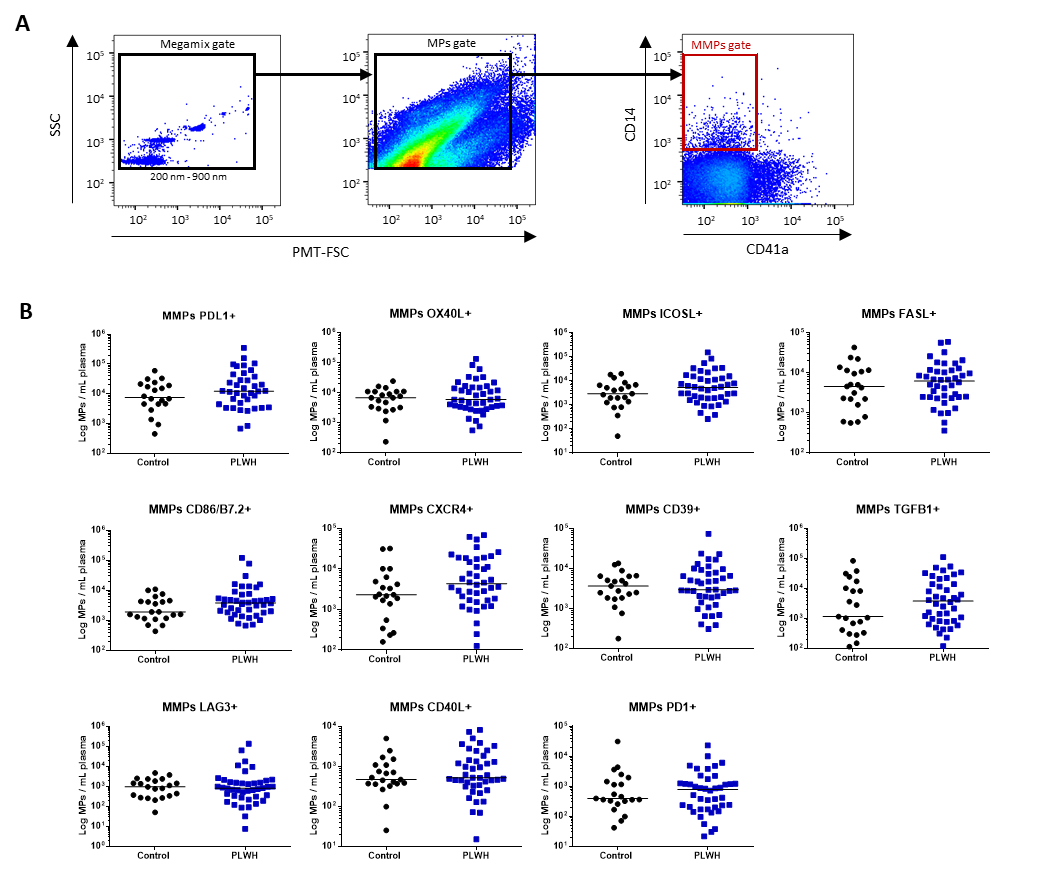
Supplemental Figure 4. Immunoregulatory molecule phenotype of MMPs from PLWH versus controls. (A)** Example of the gating strategy used in the phenotyping of CD14^+^ MMPs by flow cytometry for a control group (*n* = 21) and PLWH (*n* = 42) (21 experiments, with 2 patients and 1 control per experiment). The dot plot on the left shows the settings based on fluorescent beads for the differentiation of three particle sizes: 200, 500 and 900 nm in diameter. A dot plot for MPs is shown in the middle and a dot plot for CD14^+^ MMPs is shown on the right, for acquisition on a Fortessa flow cytometer for one representative PLWH. Flow cytometry data were analyzed with FlowJo software. **(B)** The surface expression of immunoregulatory markers on MMPs was quantified by flow cytometry on fresh plasma samples and the concentrations of MMPs expressing each of the markers were determined with Trucount tubes. The immunoregulatory markers are arranged in descending order of the number of MPs/mL of plasma expressing the marker concerned. Horizontal bars indicate the median values. *P* values were obtained in Mann-Whitney and post hoc tests.


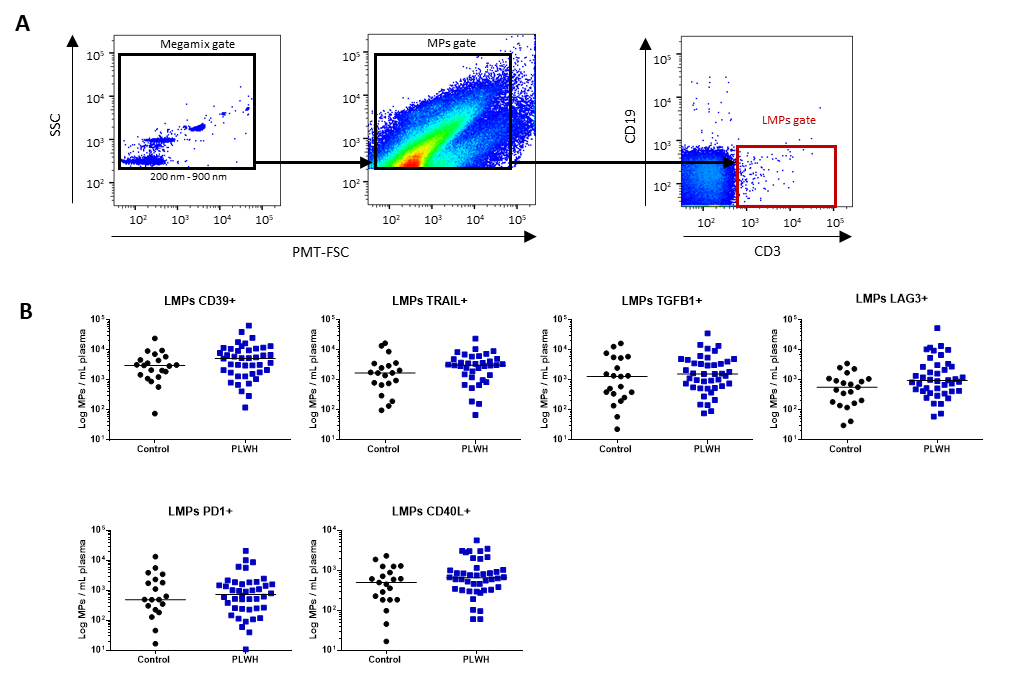


**Supplemental Figure 5. Immunoregulatory molecule phenotype of TL MPs from PLWH versus controls. (A)** Example of the gating strategy used in the phenotyping of CD3^+^ LMPs by flow cytometry for the control group (*n* = 21) and PLWH (*n* = 42) (21 experiments, with 2 patients and 1 control per experiment). The dot plot on the left shows the settings based on fluorescent beads for the differentiation of three particle sizes: 200, 500 and 900 nm in diameter. A dot plot for MPs is shown in the middle, and another, for CD3^+^ LMPs, is shown on the right, with acquisition on a Fortessa flow cytometer for one representative PLWH. Flow cytometry data were analyzed with FlowJo software. **(B)** The surface expression of immunoregulatory markers on LMPs was quantified by flow cytometry on fresh plasma samples and the concentrations of LMPs expressing each of the markers were determined with Trucount tubes. The immunoregulatory markers are arranged in descending order of the number of MPs/mL of plasma expressing the marker concerned. Horizontal bars indicate the median values. *P* values were obtained in Mann-Whitney tests and post hoc tests.

**
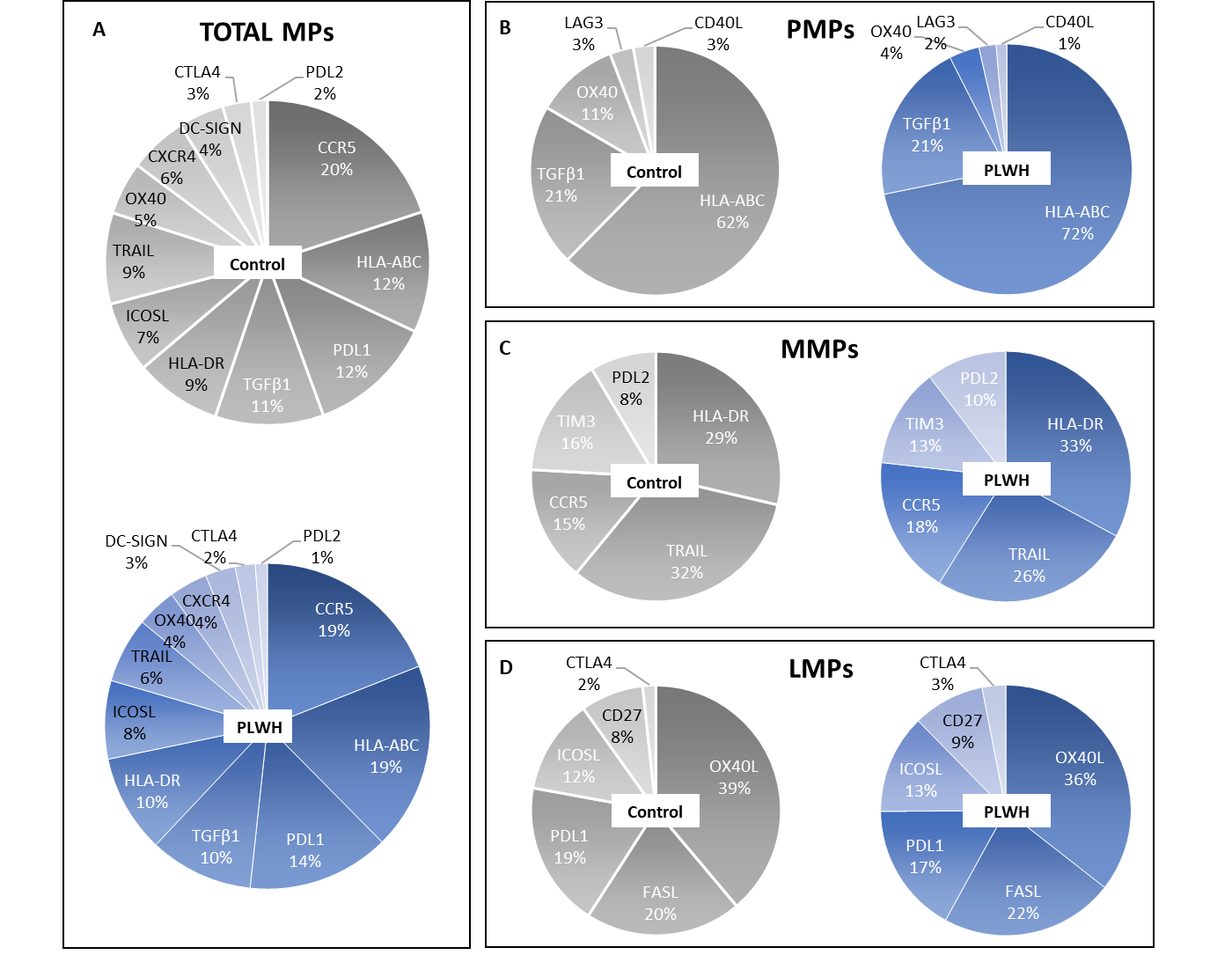
Supplemental Figure 6. Frequency of each immunoregulatory marker on MPs.** The frequencies of different immunoregulatory marker were determined from the absolute number of these MPs as a proportion of **(A)** total MPs, **(B)** PMPs, **(C)** MMPs and **(D)** LMPs. The gray and blue graphs represent the control and PLWH groups respectively.
